# Cotton Defense Induction Patterns Under Spatially, Temporally and Quantitatively Varying Herbivory Levels

**DOI:** 10.3389/fpls.2017.00234

**Published:** 2017-02-21

**Authors:** Michael Eisenring, Michael Meissle, Steffen Hagenbucher, Steven E. Naranjo, Felix Wettstein, Jörg Romeis

**Affiliations:** ^1^AgroscopeZurich, Switzerland; ^2^United States Department of Agriculture – Agriclutural Research Service, Arid Land Agricultural Research Center, MaricopaAZ, USA

**Keywords:** defense induction, *Gossypium barbadense* (cotton), *Gossypium hirsutum* (cotton), herbivory, jasmonic acid, optimal defense theory, orthostichy, terpenoids

## Abstract

In its defense against herbivores, cotton (*Gossypium* sp.) relies in part on the production of a set of inducible, non-volatile terpenoids. Under uniform damage levels, *in planta* allocation of induced cotton terpenoids has been found to be highest in youngest leaves, supporting assumptions of the optimal defense theory (ODT) which predicts that plants allocate defense compounds to tissues depending on their value and the likelihood of herbivore attack. However, our knowledge is limited on how varying, and thus more realistic, damage levels might affect cotton defense organization. We hypothesized that the allocation of terpenoids and densities of terpenoid-storing glands in leaves aligns with assumptions of the ODT, even when plants are subjected to temporally, spatially and quantitatively varying caterpillar (*Heliothis virescens*) damage. As expected, cotton plants allocated most of their defenses to their youngest leaves regardless of damage location. However, defense induction in older leaves varied with damage location. For at least 14 days after damage treatments ended, plants reallocated defense resources from previously young leaves to newly developed leaves. Furthermore, we observed a positive hyperbolic relationship between leaf damage area and both terpenoid concentrations and gland densities, indicating that cotton plants can fine-tune defense allocation. Although it appears that factors like vascular constraints and chemical properties of individual defense compounds can affect defense levels, our results overall demonstrate that induced defense organization of cotton subjected to varying damage treatments is in alignment with key assumptions of the ODT.

## Introduction

Cotton (*Gossypium* sp.) is attacked by a rich complex of arthropod herbivores and therefore possesses a large array of different inherent defense mechanisms (King et al., 1996; Hagenbucher et al., 2013a). In addition to constitutive defense traits, cotton also possesses inducible defenses, which are often systemic responses to herbivore attack (Karban and Baldwin, 1997; Hagenbucher et al., 2013a). Among the best-documented inducible cotton defense compounds is a set of biosynthetically related, non-volatile terpenoids (gossypol, hemigossypol, hemigossyplone, heliocides), which are stored in subepidermal pigment glands (Altman et al., 1990; Hagenbucher et al., 2013a). Many *in planta* and artificial diet-based studies demonstrate that cotton terpenoids provide resistance against a range of different lepidopteran herbivores (Chan et al., 1978; Stipanovic et al., 1978; Bezemer et al., 2003; Anderson and Agrell, 2005). Cotton terpenoids may also impact pest management strategies (Peterson et al., 2016) as they are known to interact with higher trophic levels (predators and parasitoids) (Du et al., 2004; Evangelista Junior et al., 2011; Correa et al., 2014; Hagenbucher et al., 2014) and affect the efficacy of genetically engineered cotton defenses (Anilkumar et al., 2009; Mészáros et al., 2011). Thus, an in-depth understanding of the factors that affect *in-planta* defense allocation dynamics in cotton is crucial for predicting and explaining insect-plant interactions in agroecosystems.

A wide range of theoretical concepts have been developed to better understand ecological and evolutionary processes that drive herbivore defense organization in plants (Stamp, 2003; Schuman and Baldwin, 2016). One of these is the optimal defense theory (ODT), which states that plant defenses are costly in terms of fitness, and that in order to optimize a plant’s fitness defense compounds will be allocated to tissue depending on the likelihood of herbivore attack and the fitness value of that tissue (McKey, 1974, 1979; Rhoades, 1979; Zangerl and Bazzaz, 1992). Thus, one of many assumptions of the ODT is that plant organs with a high fitness value such as shoots, flowers or young tissue should be well defended, whereas old tissue should contain lower defense levels (McKey, 1974, 1979; Rhoades, 1979; Stamp, 2003). The ODT as a whole is considered to be hard or even impossible to test (Rhoades, 1979). Therefore, studies often only explore certain assumptions of the ODT. In many cases leaves, which are among the best-characterized plant organs, have been used to study aspects of the ODT (Ohnmeiss and Baldwin, 2000; McCall and Fordyce, 2010; Godschalx et al., 2016). In general, young leaves are considered as having a higher photosynthetic and physiological value than old leaves (Harper, 1989; Coleman and Leonard, 1995). Studies with cotton have found that *in-planta* allocation of induced terpenoids supports the ODT because the levels of induced terpenoids in young leaves were much higher compared with old leaves (Bezemer et al., 2004; Opitz et al., 2008; Hagenbucher et al., 2013b). However, all these results were obtained under uniform damage treatments. Knowledge of how varying, and thus more realistic, damage levels might affect inducible cotton defense organization is still limited and based mainly on studies that measured terpenoid allocation indirectly via herbivore responses. Furthermore, most studies on cotton have focused on *Gossypium hirsutum*, which is economically the most relevant cotton species (PORTA4U, 2017). Other cotton species, such as *Gossypium barbadense*, the second most cultivated species remains comparatively poorly studied.

Studies on inducible plant defenses have found that undamaged leaves sharing a direct vascular connection with damaged leaves show greater defense induction compared with those that do not (Davis et al., 1991; Orians et al., 2000; Schittko and Baldwin, 2003; Ferrieri et al., 2015). Therefore, damage location may affect defense distribution in systemically inducible plants. To what extent spatially varying damage might restrict terpenoid allocation in cotton has not been addressed in detail. In addition to spatial damage variation, inducible defenses also are known to be affected by temporally varying damage levels. Induced defenses are generally activated relatively quickly, but the duration of defense relaxation is often comparatively long (Karban, 2011). Cotton and other inducible plants have indeed been observed to display increased levels of defenses in their youngest leaves for several days or weeks after damage cessation (Underwood, 1998; Anderson et al., 2001; Gutbrodt et al., 2011). With advancing time, however, young leaves decrease in value as they are replaced by newly developing foliage (Ohnmeiss and Baldwin, 2000; Barto and Cipollini, 2005; Heath et al., 2014). Plants should thus reallocate induced defenses to newly developed leaves whereas defense levels in the former youngest leaves should decrease. Knowledge is limited as to the defense allocation patterns during the relaxation phase (the period between damage cessation and when plant defenses return to pre-damage levels) (Metlen et al., 2009). For cotton, defense allocation during the relaxation phase has only been indirectly examined via herbivore responses for the very youngest leaves (Anderson et al., 2001). No information exists about detailed allocation dynamics of individual cotton terpenoids over time. A basic assumption of the ODT is that defenses are costly and that plants strive to optimize cost-related trade-offs (McKey, 1974, 1979; Rhoades, 1979). Plants should therefore be able to fine-tune their inducible defense responses in relation to damage intensity in their youngest, most valuable leaves. It has been shown that inducible plants are able to respond to different damage levels (Baldwin and Schmelz, 1994; Wold and Marquis, 1997; Mithöfer et al., 2005), but the relationship between damage intensity and individual terpenoid induction levels in the youngest leaves is not fully understood for cotton.

In this study, we exposed plants to spatial, temporal, and quantitative variations of herbivore stress. We then measured concentrations of several terpenoids and quantified the density of the terpenoid-storing producing foliar pigment glands, which are often used as a proxy for defense induction in cotton (McAuslane et al., 1997; McAuslane and Alborn, 1998; Agrawal and Karban, 2000). Specifically, we tested the following hypotheses:

(i) The concentration of induced defense compounds is highest in the youngest and thus most valuable leaves of damaged plants, regardless of the damage location on the plant;
(ii) During the relaxation phase, induced defenses are attenuated in former youngest leaves and highest in the current youngest leaves;
(iii) Plants adjust the level of induced defense compounds in their youngest leaves in response to damage intensity.

## Materials and Methods

### Plants and Insects

Cotton plants, *G. hirsutum* (Sure Grow 125, supplied by Monsanto Company, St. Louis, MO, USA) and *G. barbadense* (Deltapine 340, Olvey and Associates, Maricopa, AZ, USA) were individually grown in a climatized glasshouse (25°C, 70% RH) in 3 l plastic pots containing heat-treated, humus-rich soil and 15 g of the slow release fertilizer Manna (15% N, 7% P_2_O_5_, 15% K_2_O, Wilhelm Haug, Ammerbuch, Germany). Plants were watered as needed and fertilized weekly using 100 ml of a 10% N, 6% P_2_O_5_, 6% K_2_O solution at 10 ml l^-1^ (Manna LIN W NPK, Wilhelm Haug). For protection against glasshouse pests, all plants were enclosed in organdy cloth cages (mesh-width: 0.44 mm). All glasshouse experiments were conducted in the same climatized glasshouse.

*G. hirsutum* plants for field experiments were individually grown in Jiffy peat pellets in a glasshouse (27°C, 30% RH) in Maricopa, AZ, USA. Once the plants had fully developed cotyledons they were transplanted in an unsprayed cotton field at the USDA-ARS research station in Maricopa, AZ, USA. Each plant was marked with a plastic tag.

*Heliothis virescens*, originally obtained from Bayer Crop Science (Monheim, Germany), was reared at Agroscope in a climate chamber (25°C, 70% RH, 16 h L: 8 h D) on an artificial diet based on soy flour, wheat germ and brewer’s yeast.

### Experimental Set-Up

To induce plant defense in all glasshouse experiments, third instar *H. virescens* were caged on specified leaves using organdy cloth bags. Bags were tightly sealed around leaf petioles using cotton batting. To quantify caterpillar damage a photo was taken from damaged leaves after the caterpillar(s) were removed. The consumed leaf area was then quantified using the software ImageJ (1.48v, Schneider et al., 2012). Plants which were damaged unintentionally during the glasshouse experiments were removed from the study. Leaf samples collected for terpenoid analyses were immediately stored at –80°C for a maximum of 2 months. Each of the three glasshouse experiments as well as the field experiment described below was replicated 10–12 times and the experimental unit was an individual plant. From here forward, true leaves are abbreviated according to their position on the plant. Hence the oldest true leaf will be referred to as “L1”, the second oldest “L2” etc. A plant in the four-leaf stage would have four fully unfolded true leaves.

#### Experiment 1: Influence of Spatial Variation of Damage on Defense Allocation

*G. hirsutum* and *G. barbadense* plants in the four-leaf stage were exposed to one of four treatments in the glasshouse: (i) L1 was enclosed within an organdy cloth bag containing one *H. virescens* caterpillar, (ii) L4 was enclosed within an organdy cloth bag containing one *H. virescens* caterpillar, (iii) L1 was enclosed within an empty organdy cloth bag (control), (iv) L4 was enclosed within an empty organdy cloth bag (control). All caterpillars and bags were removed after seven days. At that time, plants had developed three additional leaves for a total of seven true leaves. The cotyledons and all available true leaves were collected and defense parameters were analyzed as described below.

#### Experiment 2: Allocation of Defense Compounds as a Function of Leaf aging

##### Glasshouse study

Plants (*G. hirsutum*) in the four-leaf stage were used in this study. The L4 of all plants was enclosed in an organdy cloth bag for the duration of the experiment. On one half of the plants a single *H. virescens* caterpillar was released on the enclosed L4, while the other half remained uninfested (control). After 7 days, the caterpillars were removed from the infested plants. Infested and uninfested plants were then divided into three groups from which leaf samples were collected either (i) immediately, (ii) after one week, or (iii) after two weeks. In all groups the L7 and the youngest leaf on the main shoot were collected, i.e., in group (i) L7 was collected, in group (ii) L7 and L10, and in group (iii) L7 and L12 were collected. Leaf samples were analyzed as described below.

##### Field study

To verify induction patterns found in the glasshouse under field conditions, the experiment was repeated between July and August 2015 in an unsprayed commercial cotton (*G. hirsutum*) field at the USDA-ARS research station in Maricopa, AZ, USA. The field was fertilized with a total of 220 kg /ha N applied over four applications of 55 kg every 3 weeks, starting with the first irrigation and ending at peak bloom. Because all *H. virescens* larvae that were used to artificially infest cotton plants in a preliminary experiment died (most likely due to high temperatures) one half of the plants in the four-leaf stage were treated with jasmonic acid (JA), a plant hormone known to induce defenses in cotton plants (Rodriguez-Saona et al., 2001) whereas the other half remained untreated (control). 250 mg of JA (≥95%, Sigma–Aldrich, Buchs, Switzerland) was dissolved in 5 ml EtOH (95%). For each treated plant, 40 μl of this solution (containing 2 mg JA) was mixed with 300 μl distilled water. This solution was then applied in single droplets to the first three true leaves and the cotyledon of each treated plant using a micro-pipette. Care was taken to ensure that no solution ran off the leaves. After 7 days the position of the youngest leaf was noted for each plant. Treated and control plants were then divided into three groups from which leaf samples were collected either (i) immediately, (ii) after 1 week, or (iii) after 2 weeks. At the date of leaf collection, the leaf that was youngest after seven days and the current youngest leaf of the main shoot were sampled, i.e., in group (i) one of the leaves L5–L7 was collected, in group (ii) one of the leaves L5–L7 and one of the leaves L8–L11, and (iii) one of the leaves L5–L7 and one of the leaves L10–L14 were collected and analyzed as described below.

#### Experiment 3: Influence of Damage Intensity and Duration on Defense Allocation

The experiment was conducted with plants (*G. hirsutum*) in the four-leaf stage. The L4 of all plants was enclosed in an organdy cloth bag for a total of 7 days and the leaf was exposed to one of five treatments: (i) one, (ii) three, (iii) nine *H. virescens* caterpillars for two days, (iv) one caterpillar for seven days, or (v) no caterpillar (uninfested, negative control). When all organdy cloth bags were removed from the plants after seven days, the youngest leaves (L7) were sampled and analyzed as described below.

### Analyzed Defense Parameters

#### Terpenoids

Between 9 and 11 mg of lyophilized leaf material from the center of each analyzed leaf was pulverized in a 2 ml Eppendorf tube, using one 3 mm tungsten carbide ball in a MM300 mixer mill (Retsch, Haan, Germany), and extracted according to the method of Benson et al. (2001). A 1 ml mixture of acetonitrile (≥99.9%, Scharlau, Barcelona, Spain), MilliQ-water and ortho-phosphoric acid (≥85%, Sigma–Aldrich) (80:20:0.1) was added to each tube. Tubes were then ultrasonicated for 3 min and centrifuged for 3 min (8 × *g*). Extracts were transferred to glass vials for analysis with a high-performance liquid chromatography (HPLC) system (1090 Series II, Hewlett-Packard, Palo Alto, CA, USA; column: Varian Polaris Amide C-18 column, 150 mm × 2.0 mm, 3 μm, equipped with a precolumn C18, 4 mm × 3.0 mm, Supelco Security Guard System). HPLC analyses followed the methodology described by Hagenbucher et al. (2013b). Gossypol was identified by comparing the retention time to that of a standard gossypol solution (gossypol from cotton seeds ≥95%, Sigma–Aldrich). The retention times of hemigossypolone, hemigossypol, heliocides B 1/4, and heliocides H1/H4 were identified based on previously published chromatograms (Stipanovic et al., 1977; Stipanovic et al., 1988; Benson et al., 2001). The identity of the terpenoids was furthermore confirmed by mass spectrometry. We were unable to confirm the identity of peaks assigned to heliocide H2/H3 with mass spectrometrical analyses. We therefore do not include heliocide H2/H3 in this study. Terpenoid concentrations were quantified in terms of gossypol equivalents (McAuslane et al., 1997).

#### Gland Density

For each analyzed leaf, the number of pigment glands was counted on photographs (Nikon D 200, objective: AF-S Micro Nikkor 105 mm 1:2.8 G ED) from 0.196 cm^2^ sized areas (using a hole template) of the leaf tip, mid and base. In cases where leaves were very small, only pictures from tip and base areas could be taken. Subsequently, the average number of glands/0.196 cm^2^ (average gland density) was calculated for each leaf. Since gland density varies with leaf size (density is higher on small leaves compared to bigger leaves of the same age, personal observation) the average gland density per leaf was standardized for leaf size by multiplying it by leaf midrib length.

### Statistical Analyses

All statistical analyses were conducted using the software R (version 3.2.3) (R Core Team, 2016).

#### Feeding Damage

Depending on the number of treatments, damage among treatments was compared using a Welch two-sample *t*-test (two treatments, experiment 1) or an Analysis of Variance (ANOVA) followed by a Tukey HSD *post hoc* test (more than two treatments, experiment 2). Control treatments (damage = 0 cm^2^) were not included in the analysis. For experiment 3, the control treatment was included in the analysis and the means were compared using Kruskal–Wallis test (package “agricolae”).

#### Experiment 1

Treatment effects on all measured defense parameters were analyzed separately for each leaf position. Because assumptions for normality and homoscedasticity were not met in all cases, Kruskal–Wallis tests were used for multiple comparisons among damage treatments followed by Holm–Bonferroni *post hoc* tests.

#### Experiment 2

Assumptions of normality and homoscedasticity were not met in any case, therefore, non-parametric tests were used to test for differences among treatments. To test for the effect of leaf age on constitutive defense parameter expression, measured defense parameters were compared among all leaves of all control treatments using Kruskal–Wallis tests. The effects of leaf age were quantified by comparing defense parameter expression among all leaves of all caterpillar-damaged treatments using Kruskal–Wallis tests followed by Holm–Bonferroni *post hoc* tests. Effects of control and damaged treatments on defense parameter expression were analyzed separately for each leaf position using Wilcoxon rank sum tests followed by Holm-Bonferroni correction.

#### Experiment 3

To analyze the relationship between the amount of leaf damage and defense parameter expression a linear and two non-linear parametric regression models describing a hyperbolic curve [two parameter single exponential rise to maximum: *y* = a(1–b^-x^) and single rectangular two parametric hyperbola: *y* = ax/(b+x)] were fitted to data sets containing either ln-transformed or non-transformed defense parameter quantities and the corresponding leaf damage in cm^2^ (package “nls2”). In order to avoid a potential confounding effect of damage duration only plants damaged for two days by two, three or nine caterpillars were included in the datasets. The fit of the models was compared within each defense parameter based on the sum of squared residuals (SS_R_) at the ln scale. Because terpenoids, which are stored in glands, are biosynthetically related, the effect of different damage treatments on defense compound production and gland densities were analyzed using multivariate analysis of variance (MANOVA) using Wilk’s lambda statistic. Differences in individual defense compounds were subsequently analyzed using ANOVA followed by Tukey HSD post-hoc tests (package “agricolae”). For analyses heliocides H1/H4 were ln-transformed to meet the assumptions of normality.

## Results

### Experiment 1: Influence of Spatial Variation of Damage on Defense Allocation

#### Gossypium hirsutum

The leaf damage caused by the caterpillars did not differ significantly between L1 (6.52 ± 0.85 cm^2^ damage) and L4 (9.53 ± 1.58 cm^2^) (Welch test; *t* = –1.68, *p* = 0.11, df = 16.88).

**FIGURE 1:**
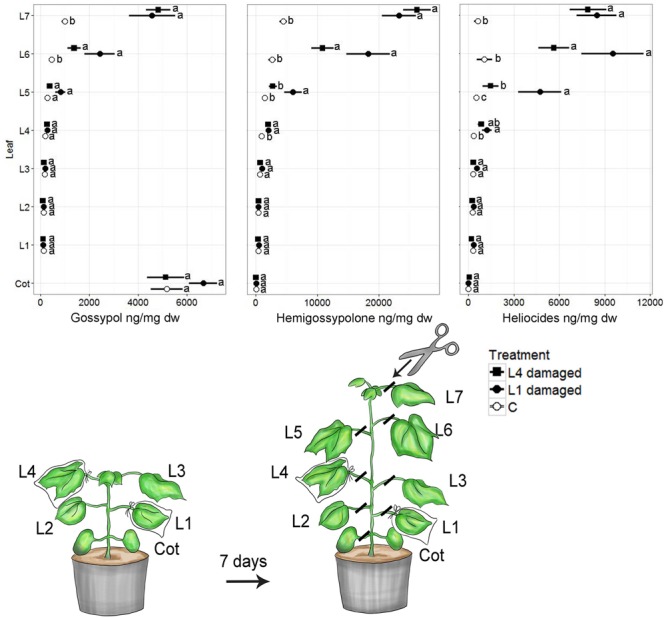
**Mean (±SE) concentrations of terpenoids in cotyledons (Cot) and fully developed true leaves (L1–L7) of *Gossypium hirsutum*.** Plants with four fully developed true leaves were infested with a single *H. virescens* larva on either the first (oldest, L1) or the fourth (youngest, L4) true leaf for 7 days. Control plants (C) were not infested. After 7 days the plants had developed an additional three leaves (L5–L7). Different letters adjacent to means indicate significant differences (*p* < 0.05) among treatments within each leaf position.

Caterpillar damage to both L1 and L4 led to significantly greater concentrations of all terpenoids in the youngest two leaves (L6, L7), compared to non-damaged control plants (Kruskal–Wallis; df = 2, all *p* < 0.001) (**Figure 1**). Plants damaged at L1 and L4 did not differ significantly in defense compound levels in L6 and L7 (Kruskal–Wallis; df = 2, all *p* > 0.12). However, except for gossypol, plants damaged at L1 showed significantly higher defense compound concentrations in L5 compared with plants damaged at L4 (*t* Kruskal–Wallis; df = 2, all *p* < 0.05). Furthermore, plants damaged on L1 showed strong trends for higher defense compound concentrations in L6 compared to plants damaged at L4. Older leaves (excl. cotyledons) had similar low levels of measured defense parameters in all treatments. Leaf gland density as a proxy for defense induction showed a distribution pattern similar to terpenoids (Supplementary Figure S1).

#### Gossypium barbadense

Leaf damage caused by the caterpillars was significantly larger on L4 (25.43 ± 2.96 cm^2^ damage) compared to L1 (13.14 ± 1.24 cm^2^) (Welch test; *t* = –3.83, *p* < 0.002, df = 13.42).

The spatial patterns of defense compound and gland densities of *G. barbadense* were similar to those in *G. hirsutum* (Supplementary Figure S2). However, the mean concentration of heliocides H1/H4 and hemigossypolone in the two youngest, induced leaves were two- to threefold lower, and gossypol levels up to four times lower in *G. barbadense* compared with *G. hirsutum*.

Whereas plants that were damaged on L1 and L4 had similar defense compound levels in L7, plants damaged at L1 had significantly higher concentrations of all terpenoids and higher gland densities in L6 compared with plants damaged on L4 (df = 2, *p* < 0.006) (Supplementary Figure S2).

#### Experiment 2: Allocation of Defense Compounds as a Function of Leaf Aging

Caterpillar damage caused to L4 did not differ among the treatment groups (damage means per treatment varied between 4.07 ± 0.62 and 5.11 ± 1.22 cm^2^) (*F*_2,28_= 0.412, *p* < 0.67).

**FIGURE 2:**
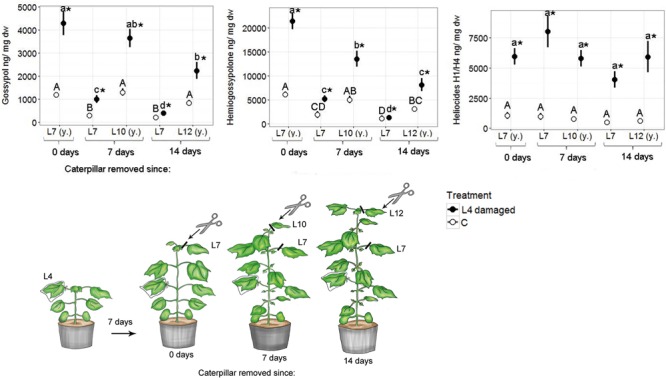
**Mean (±SE) concentrations of terpenoids in fully developed true leaves (L7, L10, L12) of *G. hirsutum*.** Plants of the four-leaf stage were infested on the L4 with one *H. virescens* larva for seven days. After 7 days the caterpillars were removed. The seventh as well as the actual youngest leaf were analyzed immediately, 7 days, or 14 days after caterpillar removal. (y) = youngest leaf at time of sampling. Control plants (C) were not damaged but sampled with the same schedule as damaged plants. Different letters above means indicate significant differences (*p* < 0.05); defense compound levels were compared among leaves of all control plants (capital letters) and leaves of all damaged plants (small letters). Within each leaf position defense compound concentrations of control and damaged leaves were compared and significant differences (*p* < 0.05) are indicated with an asterisk.

Fourteen days after caterpillar damage ceased, damaged plants still had significantly higher defense compound levels in their youngest leaves compared with the youngest leaves of control plants (**Figure 2**). The levels of gossypol and hemigossypolone were significantly lower in youngest leaves analyzed 14 days after damage ceased compared with the levels in youngest leaves analyzed 7 days or immediately after damage cessation. The level of heliocides did not significantly decrease in the youngest leaves over time. Control and damaged plants showed similar gland densities 7 days after caterpillar damage (Supplementary Figure S3).

Concentrations of gossypol and hemigossypolone strongly decreased in the induced L7 with time and were already significantly lowered 7 days after caterpillar damage ceased. In contrast, heliocide levels in induced L7 did not decrease over time and did not differ significantly from heliocide levels in current youngest leaves after 14 days. Gland densities decreased quickly in L7 (Supplementary Figure S3). Gland densities showed a strong, non-significant trend to decrease with time (Supplementary Figure S3).

Plants that were induced with JA in the field showed comparable but less pronounced patterns (Supplementary Figure S4). Seven days after induction, all plants still had significantly higher defense compound levels in their actual youngest leaves compared to youngest leaves of control plants. Such differences were still significant 14 days after induction, albeit at a much smaller scale.

#### Experiment 3: Influence of Damage Intensity and Duration on Defense Allocation

The extent of leaf damage differed significantly among different infestation treatments (Kruskal–Wallis; *H* = 49.7, df = 4, *p* < 0.001) (**Table 1**). Using Wilk’s lambda statistic, MANOVA showed a significant effect of damage treatments on defense compound production and gland densities (Λ = 0.22, *F*_16,147_= 5.87, *p* < 0.001). In general, plants infested by nine caterpillars for 2 days or one caterpillar for 7 days showed the highest amount of leaf damage and the highest average expression of individual defense parameters followed by plants damaged for two days by three or one caterpillars (**Table 1**; Supplementary Table S1).

**Table 1:** Impact of different infestation treatments on the amount of leaf damage and the induction of terpenoids.

Treatment	Leaf damage cm^2^	Gossypol ng/mg dw	Hemigossypolone ng/mg dw	Heliocides H1/H4 ng/mg dw
Control (0 cat.)	0 e	1171.65 ± 278.64 d	4813.87 ± 1171.10 b	397.91 ± 133.75 c
1 cat. for 7 days	3.91 ± 0.68 b	3799.31 ± 439.93 ab	17710.85 ± 1397.87 a	2368.81 ± 358.17 ab
1 cat. for 2 days	0.60 ± 0.06 d	2321.63 ± 340.67 cd	10791.36 ± 1744.29 ab	1992.77 ± 504.37 b
3 cat. for 2 days	2.13 ± 0.16 c	2762.27 ± 371.85 bc	12294.64 ± 1989.12 a	2640.13 ± 767.75 b
9 cat. for 2 days	5.84 ± 0.60 a	4838.01 ± 423.89 a	15808.47 ± 2746.28 a	5338.67 ± 499.46 a

*Untransformed means ± SE are shown. Means in the same column sharing the same letter are not significantly different from each other (*P* > 0.05; Tukey-HSD test). Abbreviations: cat = caterpillar, dw = dry weight.*

**FIGURE 3:**
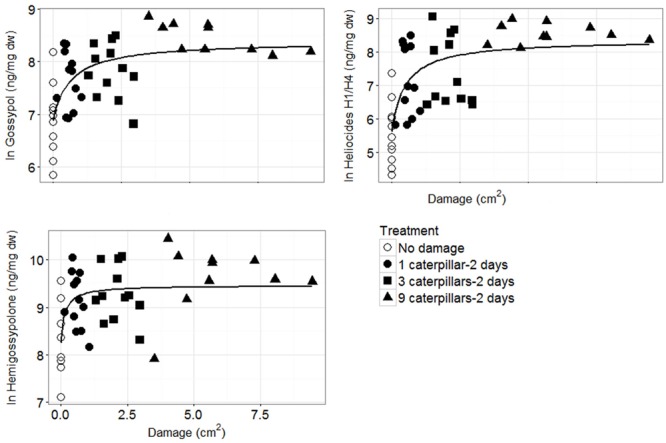
**Defense parameters (ln-transformed) of *G. hirsutum* plotted against amount of leaf damage.** The single rectangular two parameter hyperbola model: *y* = ax/(b + x) (line) achieved the best fit for all defense parameters. Note the difference in scale of the *y*-axes.

Plotting the defense parameter expression against leaf damage revealed a generally positive, but highly variable, relationship, especially at lower damage levels (**Figure 3**). The best model fit for all defense parameters was achieved by the single rectangular two parameter hyperbola model on ln-transformed defense quantities. Based on this model, defense parameter quantities correlate positively to leaf damage up to a certain level (∼2.5 cm^2^ of leaf area removed), eventually reaching a plateau (**Figure 3**, Supplementary Figure S5).

## Discussion

In general, we found that youngest cotton leaves, which are considered having a higher photosynthetic and physiological value than old leaves (Harper, 1989; Coleman and Leonard, 1995), showed the highest defense levels under spatially and temporally varying caterpillar damage. Furthermore, cotton plants were able to fine-tune defense levels in their youngest leaves with regard to damage intensity, i.e., varying herbivory levels. Thus, the organization of induced terpenoid leaf defenses in cotton confirms key assumptions of the ODT. This is in accordance with earlier studies on non-volatile terpenoids where cotton plants were exposed to uniform damage treatments (Bezemer et al., 2004; Chen et al., 2008; Opitz et al., 2008; Hagenbucher et al., 2013b). However, cotton leaf defense can also be affected by other environmental factors, e.g., nitrogen or water levels (Coviella et al., 2002; Chen et al., 2008; Luo et al., 2008; Olson et al., 2009), and so other concepts such as the carbon:nutrient balance hypothesis (Bryant et al., 1983) or the plant-stress hypothesis (White, 1969; Rhoades, 1983) may also be taken into account for a better understanding of defense allocation patterns in cotton.

Terpenoid concentrations and gland densities of the youngest leaves (L7) of damaged plants were not affected by spatially varying damage. However, we found that defense levels of slightly older leaves (L5, L6) were affected by damage location because levels of most terpenoids as well as gland densities were higher in L5 and L6 if plants were damaged on L1 compared with plants damaged on L4. Under spatially varying damage, both *G. barbadense* and *G. hirsutum*, showed similar induction patterns, indicating that our findings may be applicable across domesticated *Gossypium* species. Defense distribution within plants is often governed by the plants vascular architecture. Jones et al. (1993), for example, found that every fifth leaf of single-leader cottonwood saplings shares vascular connections. Hence, damage to L5 of a sapling leads to induction of L10 but not to induction of adjacent leaves. In many plants, translocation of leaf compounds was found to occur mainly among leaves which are in an approximate vertical row (orthostichy) (Roach, 1939; Viswanathan and Thaler, 2004). Cotton exhibits a 3/8, 2/5, or 1/3 phyllotaxis (i.e., leaves with a distance of 8, 5, or 3 leaf positions align vertically) (Hector, 1936). In our case, *G. hirsutum* and *G. barbadense* L1 and L6 leaves aligned vertically and thus exhibited a 2/5 phyllotaxis. The finding that damage on L1 leads to higher defense compound allocation to L6 might thus be partially attributed to vascular connections between L1 and L6.

Plants from the glasshouse, and to a lesser degree also from the field experiment, were able to reallocate resources for induced defense compounds (gossypol, hemigossypolone) from former youngest leaves to newly developed leaves for at least 14 days after damage termination. This finding is supported by Anderson et al. (2001) who found decreased caterpillar feeding activity for up to 14 days after cotton plants were damaged. We can only speculate why the concentrations of heliocides did not decrease within 14 days after damage cessation. Heliocides might be more stable than the other compounds and thus degrade slower. In addition, the constant concentrations of heliocides over a period of 14 days in L7 might be explained with increased conversion of hemigossypolone to heliocides in aging leaves (Bell and Stipanovic, 1976; Stipanovic et al., 1978; McAuslane et al., 1997). In contrast to terpenoid levels, damaged and control plants showed similar gland densities already 7 days after damage cessation. Opitz et al. (2008) showed that *G. hirsutum* was able to increase foliar terpenoid levels by producing additional glands but also by increasing rates of terpenoid production in already existing glands. Our data indicate that increased terpenoid levels in youngest leaves during the relaxation phase can mainly be attributed to a production of defense compounds in already existing glands, rather than the development of new glands. Gland density is therefore a less reliable proxy for measuring cotton induction during the relaxation phase then direct measures of the terpenoid levels.

We found a positive hyperbolic relationship between leaf damage area and terpenoid concentrations or gland densities. This reveals that cotton plants are able to adjust their defense allocation in youngest leaves at low degrees of herbivory, i.e., to fine-tune their induced defense allocation according to the extent of herbivory in order to reduce trade-off costs between defense expression and other processes affecting plant fitness (Rhoades, 1979; Heil, 2001). Our finding is supported by Anderson et al. (2001) who demonstrated that the reduction in caterpillar feeding activity on cotton was related to the amount of previous plant damage.

Understanding how plants respond to variable patterns of herbivory is important for understanding and predicting ecological and behavioral patterns in nature. We demonstrated that inducible defense mechanisms can enable cotton plants to respond to spatially, temporally, and quantitatively varying damage in a highly flexible way in order to defend their most valuable leaf tissue. Our results furthermore show that induced leaf defense organization of cotton subjected to varying damage treatments overall aligns with assumptions of the ODT. However, it appears that factors like chemical properties of individual defense compounds or vascular constraints can affect defense levels.

## Author Contributions

ME, MM, SH, SN, and JR planned and designed the experiment, ME and SN performed the experiments, ME and FW performed the chemical analyses ME and SN analyzed the data statistically, ME, MM, SH, SN, FW, and JR wrote the manuscript.

## Conflict of Interest Statement

The authors declare that the research was conducted in the absence of any commercial or financial relationships that could be construed as a potential conflict of interest.
