# Supplementary material for: Cotton Defense Induction Patterns Under Spatially, Temporally and Quantitatively Varying Herbivory Levels
Source: Front Plant Sci. 2017 Feb 21;8:234. doi: 10.3389/fpls.2017.00234 (PMC5318428; doi:10.3389/fpls.2017.00234)
Supplement: Supplementary file 1 [file Data_Sheet_1.docx]

Supplementary Material

**Cotton Defense Induction Patterns Under Spatially, Temporally and Quantitatively Varying Herbivory Levels**

**Michael Eisenring^1^, Michael Meissle^1^, Steffen Hagenbucher^1^, Steven E. Naranjo^2^, Felix Wettstein^1^, Jörg Romeis^1*^**

^1^ Agroscope, Zurich, Switzerland
^2^ USDA-ARS, Arid-Land Agricultural Research Center, Maricopa, Arizona USA

*** Correspondence**: joerg.romeis @agroscope.admin.ch

# Supplementary Figures and Tables


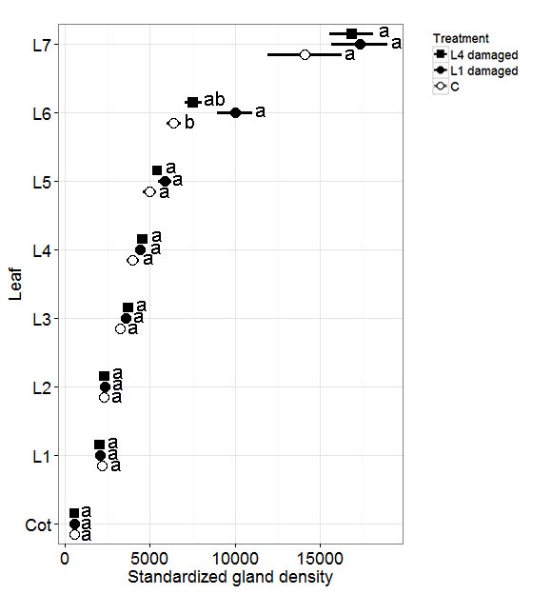


**Fig. S1** Mean (±SE) gland densities (standardized for midrip length) of cotyledons (cot) and fully developed true leaves (L1-L7) of *G. hirsutum*. Plants with four fully developed true leaves were infested with a single *H. virescens* larva on either the first (oldest, L1) or the fourth (youngest, L4) true leaf for seven days. Control plants (C) were not infested. After seven days the plants had developed an additional three leaves (L5-L7). Different letters adjacent to means indicate significant differences (p<0.05) among treatments within each leaf position.


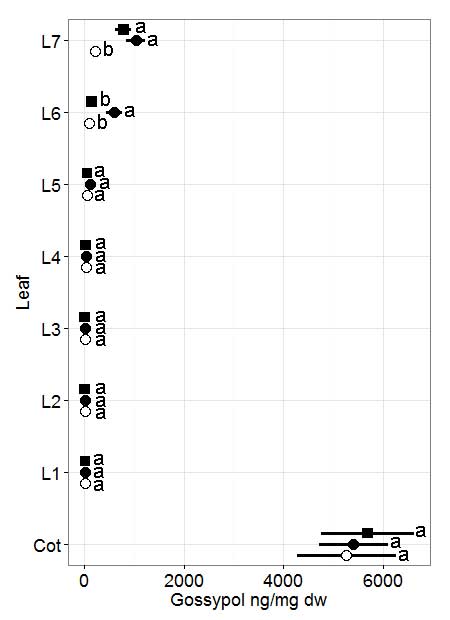

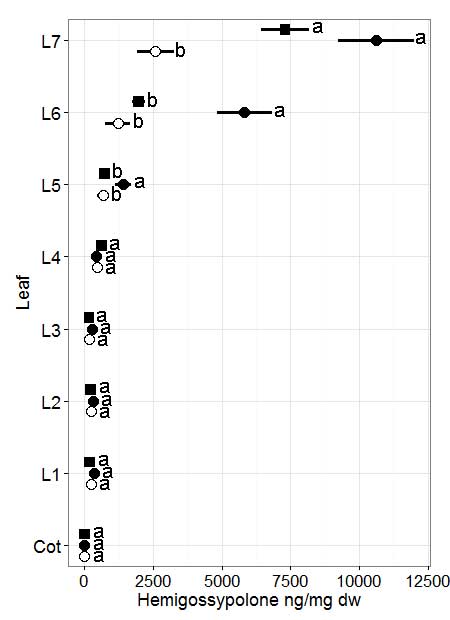

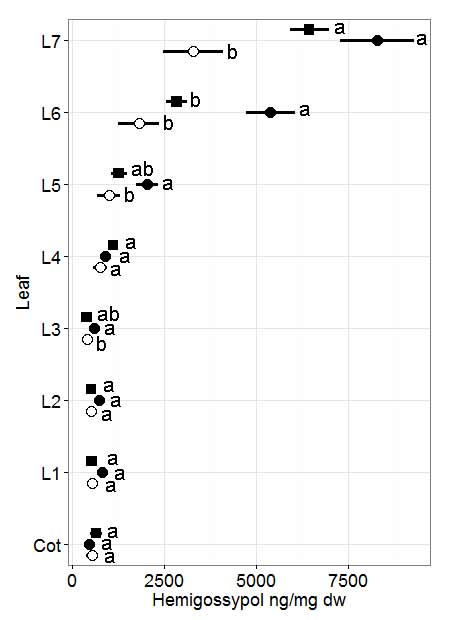

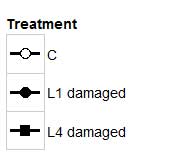

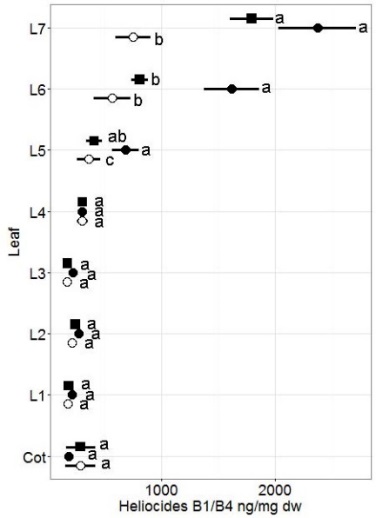

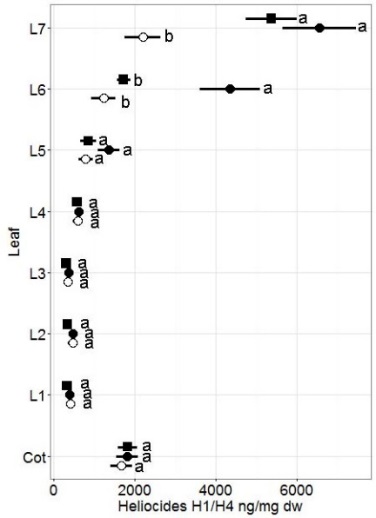

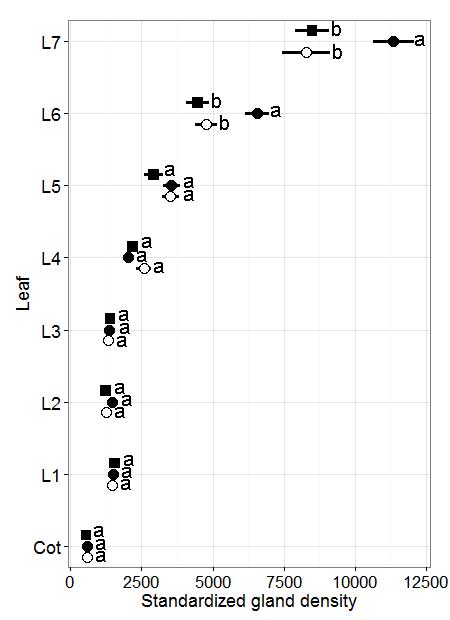


**Fig. S2** Mean (±SE) concentrations of terpenoids and standardized gland densities of cotyledons (cot) and fully developed true leaves (L1-L7) of *G. barbadense*. Plants with four fully developed true leaves were infested with a single *H. virescens* larva on either the first (oldest, L1) or the fourth (youngest, L4) true leaf for seven days. Control plants (C) were not infested. After seven days the plants had developed an additional three fully developed true leaves (L5-L7). Different letters above means indicate significant differences (p<0.05) among treatments within each leaf position.


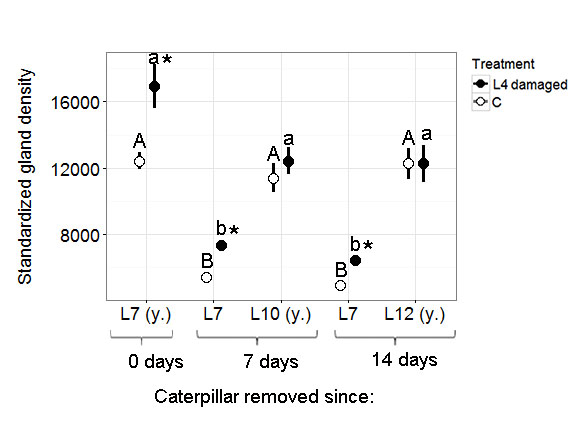


**Fig. S3** Mean (±SE) standardized gland densities of fully developed true leaves (L7, L10, L12) of *G. hirsutum*. Plants of the four-leaf stage were infested on the L4 with one *H. virescens* larva for seven days. After seven days the caterpillars were removed. The seventh as well as the actual youngest leaf were analyzed immediately, 7 days or 14 days after caterpillar removal. Control plants (C) were not damaged but sampled at the same schedule as damaged plants. (y.)=actual youngest leaf. Different letters above means indicate significant differences (p<0.05). Defense compound levels were compared among leaves of all control plants (capital letters) and leaves of all damaged plants (small letters). Within each leaf position defense compound concentrations of control and damaged leaves were compared and significant differences (p<0.05) are indicated with an asterisk.


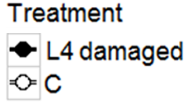


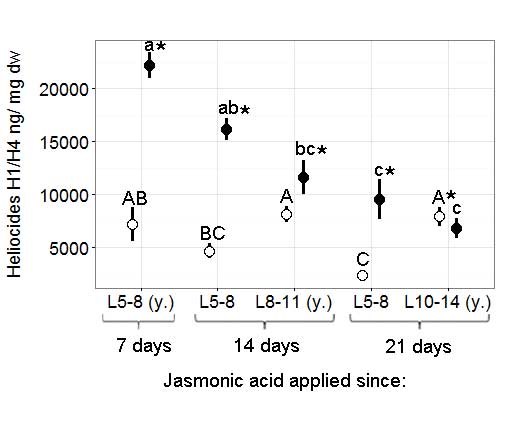

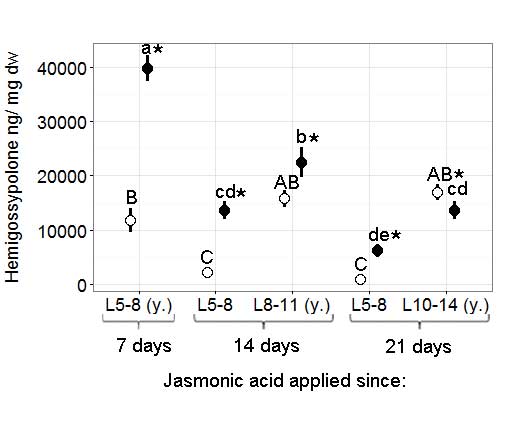

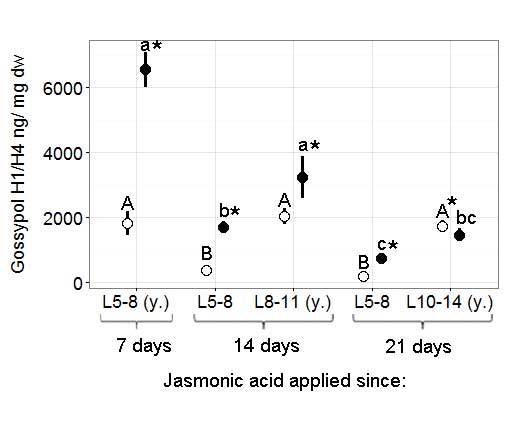

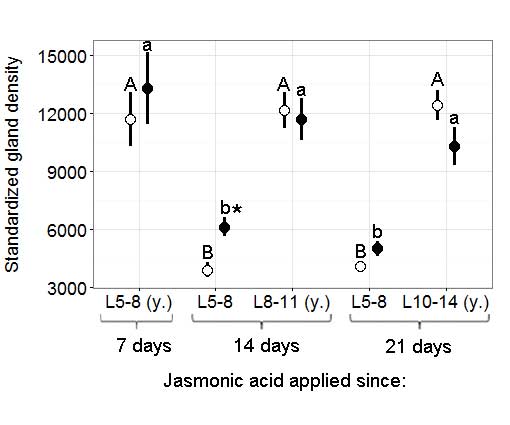


**Fig. S4** Mean (±SE) concentrations of terpenoids and standardized gland densities of fully developed true leaves (L5-L14) of *G. hirsutum* grown in the field. Plants of the four-leaf stage were induced with 40 µl of a solution containing 2mg Jasmonic acid (JA). Seven days after the JA treatment the position of the youngest leaf was noted for each plant (L5-L8). Either 7, 14 or 21 days after the JA treatment, the leaf that was youngest after seven days and the new youngest leaf of the main shoot that developed over time were analyzed. Control plants (C) were not induced but sampled at the same schedule as induced plants. (y.)=actual youngest leaf. Different letters above means indicate significant differences (p<0.05), within each JA application date; defense compound levels were compared among leaves of all control plants (capital letters) and leaves of all damaged plants (small letters). Within each leaf position defense compound concentrations of control and damaged leaves were compared and significant differences (p<0.05) are indicated with an asterisk.


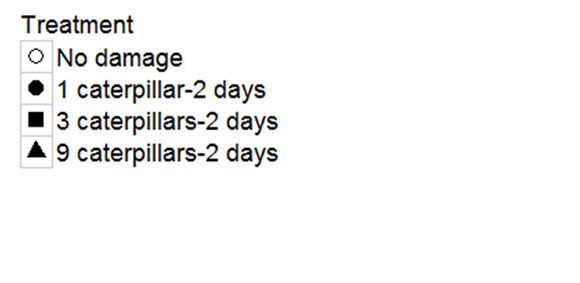

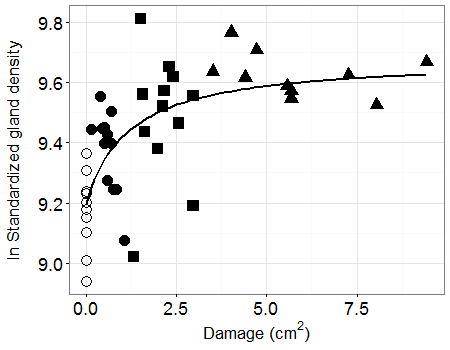


**Fig. S5** ln-transformed standardized gland densities plotted against amount of leaf damage using a single rectangular two parameter hyperbola model. Y=ax/(b+x) (line).

**Table S1** Impact of different infestation treatments on the amount of leaf damage and standardized (std.) gland density: Untransformed means ±SE are shown. Means in the same column sharing the same letter are not significantly different from each other (P> 0.05; Tukey-HSD test). Abbreviations: cat= caterpillar.

| **Treatment** | **Leaf damage cm^2^** | **Std. gland density** |
| --- | --- | --- |
| **Control (0 cat.)** | 0 **e** | 9701.06±350.14 **d** |
| **1 cat. for 7 days** | 3.91±0.68 **b** | 15940.42±824.92 **a** |
| **1 cat. for 2 days** | 0.60±0.06 **d** | 11849.83±440.25 **cd** |
| **3 cat. for 2 days** | 2.13±0.16 **c** | 13372.94±755.23 **bc** |
| **9 cat. for 2 days** | 5.84±0.60 **a** | 15175.37±359.76 **ab** |
